# Lensless Three-Dimensional Quantitative Phase Imaging Using Phase Retrieval Algorithm

**DOI:** 10.3390/jimaging6090099

**Published:** 2020-09-20

**Authors:** Vijayakumar Anand, Tomas Katkus, Denver P. Linklater, Elena P. Ivanova, Saulius Juodkazis

**Affiliations:** 1 Optical Sciences Center and ARC Training Centre in Surface Engineering for Advanced Materials (SEAM), Faculty of Science, Engineering and Technology, Swinburne University of Technology, Hawthorn, VIC 3122, Australia; tkatkus@swin.edu.au; 2 Department of Physics, RMIT University, GPO Box 2476, Melbourne, VIC 3001, Australia; denver.linklater@rmit.edu.au (D.P.L.); elena.ivanova@rmit.edu.au (E.P.I.); 3 Tokyo Tech World Research Hub Initiative (WRHI), School of Materials and Chemical Technology, Tokyo Institute of Technology, 2-12-1, Ookayama, Meguro-ku, Tokyo 152-8550, Japan

**Keywords:** quantitative phase imaging, phase retrieval, three-dimensional imaging, lensless imaging, computational optics, digital imaging, holography

## Abstract

Quantitative phase imaging (QPI) techniques are widely used for the label-free examining of transparent biological samples. QPI techniques can be broadly classified into interference-based and interferenceless methods. The interferometric methods which record the complex amplitude are usually bulky with many optical components and use coherent illumination. The interferenceless approaches which need only the intensity distribution and works using phase retrieval algorithms have gained attention as they require lesser resources, cost, space and can work with incoherent illumination. With rapid developments in computational optical techniques and deep learning, QPI has reached new levels of applications. In this tutorial, we discuss one of the basic optical configurations of a lensless QPI technique based on the phase-retrieval algorithm. Simulative studies on QPI of thin, thick, and greyscale phase objects with assistive pseudo-codes and computational codes in Octave is provided. Binary phase samples with positive and negative resist profiles were fabricated using lithography, and a single plane and two plane phase objects were constructed. Light diffracted from a point object is modulated by phase samples and the corresponding intensity patterns are recorded. The phase retrieval approach is applied for 2D and 3D phase reconstructions. Commented codes in Octave for image acquisition and automation using a web camera in an open source operating system are provided.

## 1. Introduction

Quantitative phase imaging (QPI) techniques enable the measurement of thickness and refractive index variations in optically transparent objects, and have been widely used to study unstained biological samples, which modulate the phase but not the amplitude [1,2,3,4,5,6,7,8,9,10,11,12,13,14,15]. A wide variety of techniques, such as Schlieren method [4], Zernike phase contrast method [5], tomography [6], interference microscopy [7,8,9] and digital holography [10] can be applied for QPI applications. Out of the above methods, some techniques are preferred due to their easier implementation and improved performance. For instance, the phase contrast microscope invented by Frits Zernike in 1933 consisted of annular illumination, phase and amplitude modulating ring elements in a sequence that reduced the background light and highlighted the phase profile with an improved contrast [5]. This was widely adopted later for phase imaging tasks. Later, a modified version of the phase contrast microscope called a differential interference contrast microscopy was developed by Georges Nomarski nearly two decades later [11]. In this technique, two orthogonally polarized, mutually coherent and spatially separated light waves are interfered with, and the relative phase differences are imaged. In subsequent studies, interferometry and digital holography techniques evolved into reliable phase imaging methods [12,13,14]. In most of the interference based phase imaging applications, a coherent light source is split into two: one of the beams passes through the sample, while the other beam is used as a reference. The resulting interferogram is processed to extract the phase distribution of the object. Multimodal imaging methods involving the measurement of both amplitude and phase have been developed, but require a dual beam approach [15,16], coded aperture methods [17,18], Lloyd’s mirror [19] and low-coherence two beam holography techniques [20]. In all the above techniques, many optical components such as lenses, prisms, beam splitters, polarizers, etc., are needed for beam splitting, combining and for creation of interference patterns.

In 2004, a phase contrast imaging technique was introduced [21], in which collimated laser light was modulated by a phase sample and the corresponding intensity pattern was recorded. The well-known Gerchberg–Saxton algorithm (GSA) [22,23,24] was implemented to retrieve the phase of the object. This mode of imaging is one of the most economical versions available for phase contrast imaging applications. Later, multiple wavelengths were used to improve the convergence of the phase retrieval algorithm [25]. In all the above studies, phase imaging in only a single plane was demonstrated. With the developments in smart phone devices, the lensless imaging method was converted into highly compact versions [26,27,28,29,30]. Furthermore, the technique evolved into shadow imaging [26,28], fluorescence imaging [30], and learning-based imaging [31]. Recent studies have shown the possibility of employing low coherent light for QPI. The topic has been extensively reviewed [32], but there are not many tutorials available on this topic.

In this manuscript, we demonstrate a lensless single camera shot 3D QPI technique using incoherent light. A phase retrieval algorithm has been implemented with accurate scaling and sampling conditions, such that the algorithm converges within a few iterations to the optimal reconstruction, enabling the real time monitoring of live biological specimens. The manuscript consists of seven sections. In the second section, the methodology and theoretical studies are presented. The third section describes the simulation and computational procedure. The simulative studies with different types of samples are presented in the fourth section. In the fifth section, the experiments and results are presented. The results are discussed in the sixth section. The summary and conclusion are presented in the final section. Five supplementary materials are provided, which consists of the original commented codes in Octave for simulation and real experiments with the automation of image sensor and phase retrieval. We believe that the presented tutorial will highly benefit researchers who begin research in the area of QPI.

## 2. Methodology

**Figure 1:**
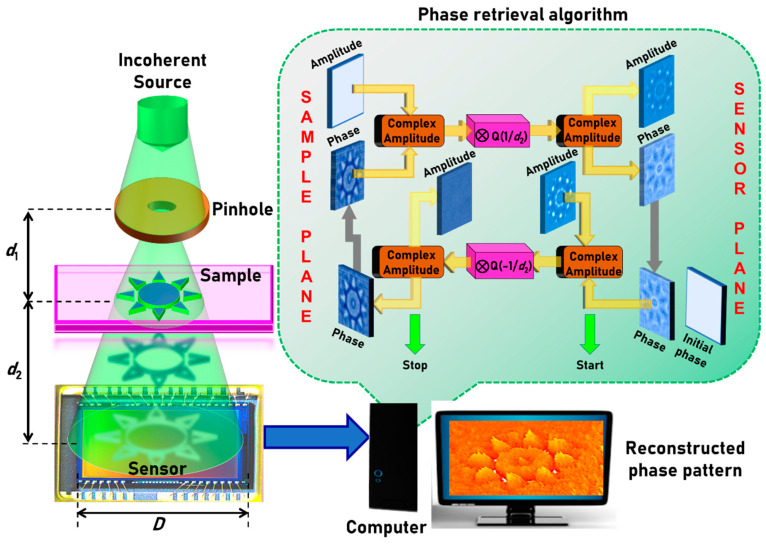
Optical configuration of lensless incoherent quantitative phase imaging (QPI) system and the schematic of the phase retrieval algorithm.

The optical configuration of a basic lensless QPI using phase retrieval algorithm is shown in Figure 1. The illumination system consists of a light emitting diode and a pinhole. The light diffracted from the pinhole is incident on the sample located at a distance of *d*_1_ from it. The light modulated by the sample is recorded by an image sensor located at a distance of *d*_2_ from the sample. The complex amplitude incident on the sample is given as $C_{1}\sqrt{I_{o}}Q\left(1/d_{1}\right)$ and after the sample it is $C_{2}\sqrt{I_{o}}Q\left(1/d_{1}\right)\exp\left[-j\Phi_{s}\left(x,y\right)\right]$, where *C*_1_ and *C*_2_ are complex constants, $\sqrt{I_{o}}$ is the amplitude of the point object generated by the pinhole, *Q*(1/*d*_1_) is the quadratic phase factor given as $Q\left(b\right)=\exp\left[j\pi bR^{2}/\lambda \right]$, where $R={\left(x^{2}+y^{2}\right)}^{1/2}$ and $\Phi_{s}\left(x,y\right)=\frac{2\pi t\left(x,y\right)}{\lambda }\left(n-1\right)$, where *t*(*x,y*) and *n* are the thickness function and refractive index of the sample, respectively. In this case, it is assumed that *n* is constant for simplicity, but it is possible that both *n* and *t* can simultaneously vary across the sample and cannot be separated without additional measurements. The complex amplitude at the image sensor is given by $C_{2}\sqrt{I_{o}}Q\left(1/d_{1}\right)\exp\left[-j\Phi_{s}\left(x,y\right)\right]⨂Q\left(1/d_{2}\right)$, where ‘⨂’ is a two-dimensional convolutional operator. The intensity distribution recorded by the image sensor can be given as ${\left|C_{2}\sqrt{I_{o}}Q\left(1/d_{1}\right)\exp\left[-j\Phi_{s}\left(x,y\right)\right]⨂Q\left(1/d_{2}\right)\right|}^{2}$. The phase of the sample is calculated using an iterative GSA [22]. There are two planes of interest: sample plane and sensor plane. It is assumed that the sample introduces only phase modulation and no amplitude modulation. For smaller distances, a spherical phase factor given by S[R(d)]=exp[j2πR(d)/λ], where $R\left(d\right)={\left(x^{2}+y^{2}+d^{2}\right)}^{1/2}$ is needed. The schematic of the GSA is shown as an inset in Figure 1. Unlike lens-based imaging techniques, lensless systems’ resolution limit is the pixel size of the sensor and the resolution differences between the maximum limit achievable (λ/2), and the pixel size is often bridged using pixel super resolution techniques [28,29]. The magnification of the system based on geometric projection is given as *M* = (1 + *d*_2_/*d*_1_), and the numerical aperture (NA) is given as *D*/2(*d*_1_ + *d*_2_), where *D* is the diameter of the image sensor.

In two beam interference-based phase measurements, the amplitude and phase at the image sensor are known and so the complex amplitude at the sample plane can be directly calculated, either by a back propagation or a Fourier transform of the complex amplitude recorded at the sensor plane. In the interferenceless-phase retrieval approaches, a part of the information, i.e., the phase information at the sensor plane, is not available. The amplitude at the sensor plane is available and the amplitude information at the sample plane is assumed or known. The algorithm begins with a complex amplitude constructed with the square root of the intensity function recorded by the image sensor as the amplitude function, and an initial guess uniform phase or random phase at the sensor plane. However, previous studies indicated a faster convergence when the initial guess phase was uniform [21]. This complex amplitude is propagated to the sample plane by convolving it with S[−R(d)]. The resulting complex amplitude’s amplitude is replaced by a uniform function, while the resulting phase is the phase of the sample which is carried on to the next iteration. This modified complex amplitude is forward propagated by convolution with S[R(d)] and so on. During every back and forth propagation, the amplitude functions were replaced by the measured and assumed functions, while the obtained phase functions were transferred as they are. By this process, the two phase functions gradually converge to satisfy the amplitude functions at the two planes. The entire process is iterated until the phase pattern is generated with a minimum error, which is measured by comparing the reconstructed phase pattern with the original phase pattern. There are numerous ways to quantify the error in the reconstructed phase. In this case, an error function defined by a cross-correlation between the original phase pattern and the reconstructed phase pattern is used for quantifying the similarity between the two. The correlation function is defined as $C={\left|\Phi_{s}\left(x,y\right)*\Phi_{r}\left(x,y,p\right)\right|}^{2}$, where $\Phi_{r}\left(x,y,p\right)$ is the phase pattern reconstructed from the phase retrieval algorithm after *p* iterations and ‘∗’ is a 2D correlation operator [33,34] with a phase-only filter [35]. Once *C* (*x* = 0, *y* = 0) reaches a stable value, the iteration is stopped. The maximum value possible is when the cross-correlation reaches the autocorrelation value, i.e., when $C={\left|\Phi_{s}\left(x,y\right)*\Phi_{s}\left(x,y\right)\right|}^{2}$. There are other alternative cost functions, such as SSIM [36], entropy [37] and mean-squared error [38] which could be used in place of the cross-correlation. Entropy and SSIM require a reference image, while entropy is a blind evaluator.

## 3. Computational Procedure

The computer simulation begins with a sampling of the planes of interest. The length and breadth of the matrices are set to match twice the image sensor’s length *N*_1_ and breadth *N*_2_ in pixels, and the pixel size Δ of the sensor is set as the sampling period for the simulation. The pseudocode for the entire process is shown in Table 1. The first step involves the definition of the computational space, sampling, meshgrid and wavelength. In the next step, the amplitude and phase matrices at the sample and sensor planes are constructed. The intensity pattern recorded by the sensor with a size of *N*_1_ × *N*_2_ is normalized and the square root values are calculated, and the matrix is zero padded to reach a matrix size of 2*N*_1_ × 2*N*_2_ to be the amplitude matrix *A*_1_ at the sensor plane. The phase matrix *P*_1_ at the sensor plane is set to be a constant for all pixels and the complex amplitude *C*_1_ is constructed using *A*_1_ and *P*_1_. The amplitude matrix *A*_2_ at the sample plane is constructed by assigning all the pixels a value of 1. The phase retrieval algorithm is executed with a *for* loop and begins at the sensor plane with a complex amplitude *C*_1_. The complex amplitude from the sensor plane is propagated to the sample plane by a convolution realized by three Fourier transform operations, given as $C_{n}⨂S={ℑ}^{-1}\left[ℑ\left(C_{n}\right)\times ℑ\left(S\right)\right]$ [38]. At the sample plane, the phase matrix was carried on while the amplitude matrix is replaced by *A*_2_. The phase at the sample plane is extracted after several iterations, when the correlation coefficient *C* (*x* = 0, *y* = 0) does not vary beyond a threshold value.

## 4. Simulative Studies

**Figure 2:**
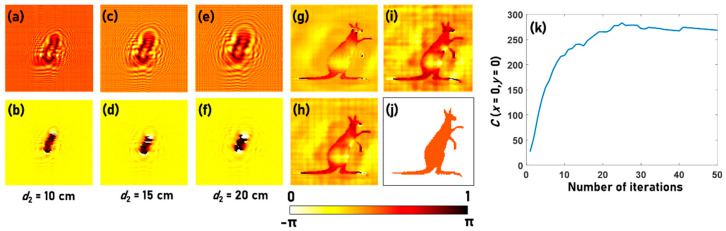
Amplitude simulated at (**a**) *d*_2_ = 10 cm, (**c**) *d*_2_ = 15 cm and (**e**) *d*_2_ = 20 cm. Phase simulated at (**b**) *d*_2_ = 10 cm, (**d**) *d*_2_ = 15 cm and (**f**) *d*_2_ = 20 cm. The phase retrieved after (**g**) 2, (**h**) 10 and (**i**) 50 iterations and the (**j**) original phase. (**k**) Plot of *C* (*x* = 0, *y* = 0) as a function of number of iterations.

The simulative studies were carried out for a wavelength *λ* = 530 nm, *d*_1_ = 200 cm and *d*_2_ = 20 cm. Five test objects are considered for simulative studies. The first object is a Kangaroo phase object with a step size of *t* = 530 nm and an index of refraction *n* = 1.5. The Kangaroo phase object is used for the first type of simulation studies. In this study, the object is illuminated by the light diffracted from a point object located at a large distance, and so the phase of the incident light is closer to a plane wave. The amplitude and phase simulated using Fresnel propagation after the sample plane at distances of 10 cm, 15 cm and 20 cm are shown in Figure 2a–f. The intensity distribution simulated at *d*_2_ = 20 cm is used in the phase retrieval algorithm. The reconstructed phase patterns after 2, 20, and 50 iterations are shown in Figure 2g–i respectively. The reference phase image is shown in Figure 2j. The plot of the correlation function as a function of the number of iterations is shown in Figure 2k. The initial phase is selected as one in this case. The performance of the algorithm is highly sensitive to the initial guess phase. The computational codes for simulation in Octave are given in Supplementary Material 1. The simulation conditions were quite direct with a nearly plane wavefront at the input. If the incident wavefront is spherical with a smaller radius of curvature, then it is necessary to optimize the distance *d*_2_ to compensate the effect of the spherical phase. The appropriate propagation distance for obtaining the best reconstruction is at $d_{2}^{\prime }\approx {\left(\frac{1}{d_{2}}-\frac{1}{d_{1}}\right)}^{-1}$ within the region of paraxial approximation. For instance, when *d*_1_ = 200 cm and *d*_2_ = 20 cm, the optimal propagation distance *d*_2_′ is 22.2 cm, which could be tested in the Octave codes provided in the Supplementary Material 1.

**Figure 3:**
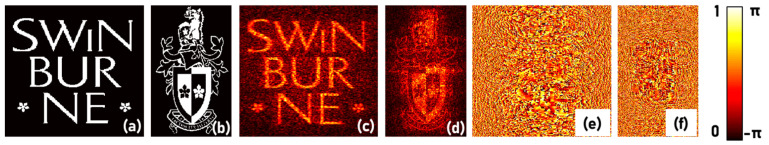
Image of the test objects (**a**) Swinburne logo at the object plane and (**b**) Swinburne emblem at the sensor plane. Reconstructed amplitude at (**c**) the object plane and (**d**) the sensor plane. Retrieved phase at (**e**) the object plane and (**f**) the sensor plane.

**Figure 4:**
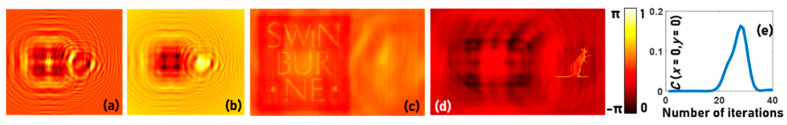
(**a**) Amplitude and (**b**) phase of the optical field at the sensor plane. Phase reconstruction by the phase retrieval algorithm at (**c**) *d*_2_ = 20 cm and (**d**) *d*_2_ = 40 cm. (**e**) Plot of the correlation coefficient as a function of number of iterations.

**Figure 5:**
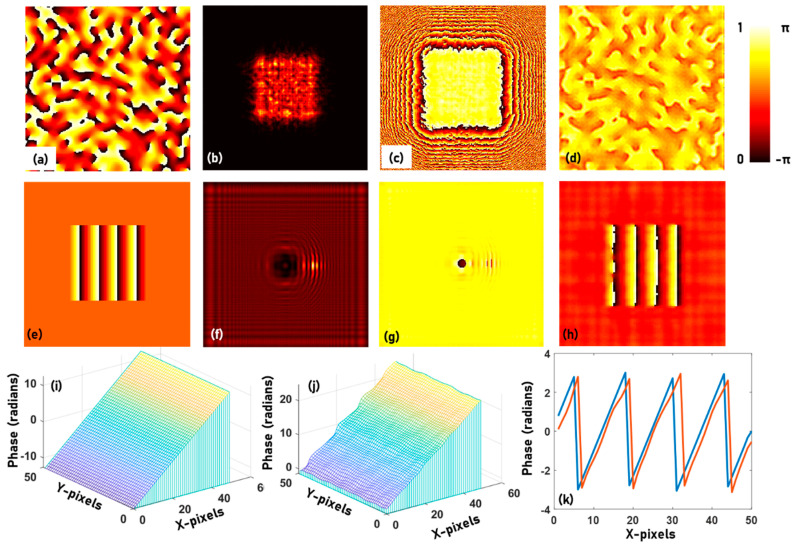
(**a**) Scattering layers synthesized using Fourier Gerchberg–Saxton algorithm (GSA) with a scattering ratio *σ* = 0.12. (**b**) Amplitude and (**c**) phase of the field at the sensor plane and (**d**) reconstructed phase after 20 iterations. (**e**) modulo-2π representation of a linear phase with a maximum phase of 8π. (**f**) Amplitude and (**g**) phase of the field at the sensor plane and (**h**) reconstructed phase after 20 iterations. (**i**) Original phase at the sample plane. (**j**) Reconstructed, unwrapped phase at the sensor plane. (**k**) Comparison of modulo-2π phase profile of the original and reconstructed phase patterns.

The next two objects, namely the logo and emblem of Swinburne, are binary amplitude objects at the sample plane and the sensor plane respectively. The phase patterns are calculated using the phase retrieval algorithm at the sample plane and the sensor plane respectively. The image of two objects, the corresponding amplitude and phase patterns at the sample and sensor planes after 200 iterations are shown in Figure 3a–f respectively. The Octave code for simulating the optical fields and phase retrieval is given in Supplementary Material 2. A thick object is constructed using the two thin phase objects made up of the Kangaroo phase object and the Swinburne logo, with a step phase difference of π with respect to the background. The distance between the two objects Δ*d* = 20 cm, *d*_1_ = 200 cm and *d*_2_ = 20 cm. The Octave code for constructing the thick object using two thin objects is given in the Supplementary Material 3. The images of the amplitude and phase at the sensor plane are shown in Figure 4a,b respectively. The image reconstruction after 10 iterations executed for *d*_2_ = 20 cm and 40 cm are shown in Figure 4c,d respectively. The presence of the background phase from the other object affects the convergence of the algorithm, as shown in Figure 4e, and so it is necessary to carefully observe the reconstructions with the number of iterations. The fourth object is a complex object consisting of a scattering layer with 200 × 200 pixels size, with a scattering ratio *σ* = 0.12, synthesized using a Fourier GSA as described in [39]. The amplitude around the scattering layer is zero. The phase image of the scattering layer, the amplitude and phase at the sensor plane and the reconstructed phase image after 20 iterations are shown in Figure 5a–d respectively. The simulation is repeated for a linear phase with a maximum value of 8π, surrounded by a constant phase of zero but unit amplitude. The image of the linear phase with modulo-2π phase modulation, the amplitude and phase at the sensor are shown in Figure 5e–g respectively. The reconstructed phase is shown in Figure 5h. The original phase profile is shown in Figure 5i. The unwrapped reconstructed phase profile is shown in Figure 5j. The simulation can be carried out using one of the Supplementary Materials 1–3 and the phase unwrapping can be achieved by the *unwrap* function in a *for* loop of Octave. The modulo-2π phase profiles of the original and reconstructed phase patterns are compared in Figure 5k. The performances of the GSA under different computational configurations have been studied [40]. The formation of greyscale objects without and with modulo-2π phase structures have been discussed in [41].

## 5. Experiments and Results

**Figure 6:**
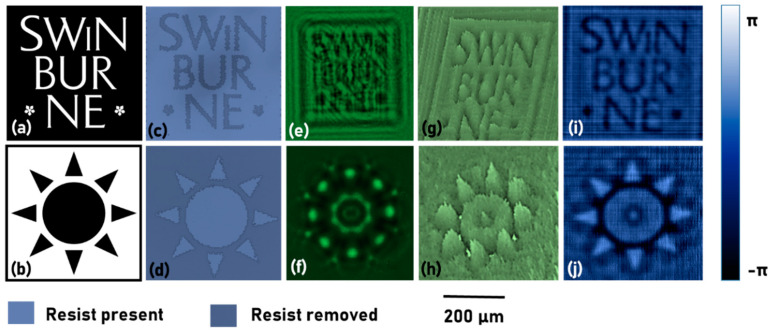
Images of the two test objects (**a**) Swinburne logo and (**b**) Star object. Optical microscope images of (**c**) Swinburne logo and (**d**) Star object. Intensity pattern recorded by the image sensor for (**e**) Swinburne logo and (**f**) Star object. Phase map generated by the phase retrieval algorithm after two iterations for (**g**) Swinburne logo and (**h**) Star object. The phase images generated by the phase retrieval algorithm after two iterations for (**i**) Swinburne logo and (**j**) Star object.

The test samples for the study were fabricated using electron beam lithography (EBL; Raith 150^2^, Dortmund, Germany) [42,43,44] in PMMA 950K(MicroChemicals GmbH Nicolaus-Otto-Str. 39 D-89079, Ulm, Germany) resist on indium tin oxide (ITO) glass substrates with 1.1 mm thickness. Two samples, namely Swinburne object and Star object, were fabricated with positive (resist removed) and negative (resist present) configurations respectively. The thickness *t* of PMMA resist was about 800 nm, and its refractive index is closely matching that of glass. The developed patterns after EBL exposure represent mostly phase structures (a jump in PMMA thickness) which were used for the optical imaging experiments. An experimental set up based on Figure 1 is built using a LED (*λ_c_* = 530 nm, FWHM = 33 nm) and a pinhole with a diameter of 100 µm. The distances were *d*_1_ = 10 cm and *d*_2_ = 5 cm. The experiment was first carried out using the single thin samples, one after the other. The images of the intensity patterns were recorded by the image sensor (Thorlabs DCU223M, 1024 × 768 pixels, pixel size = 4.65 μm). The optical microscope images of the two objects, the intensity patterns recorded using the lensless imaging system and the phase images reconstructed using the phase retrieval algorithm, are shown in Figure 6. The wavelength value of 530 nm and a pixel size of 4.65 µm were used in the phase retrieval algorithm. The propagators were synthesized for *d* = 5 cm, but the best reconstruction was obtained for *d* = 6.5 cm. The difference in the values of *d* can be caused by a range of factors, such as the spherical phase of the incident light, errors in distance measurements, the thickness of glass, etc. Since there can be only one value of the distance for every axial plane, in the first run, the optimal distance which resulted in the best focus was estimated. Later, the same distance was used for phase retrieval for objects located in the same plane. The phase maps and images shown in Figure 6 match exactly with the positive and negative resist profiles of the Swinburne logo object and the star object respectively, after fabrication. The number of iterations, in this case, is only two, making the method viable for the real-time monitoring of biological events. An open source software, Octave (version 4.4.1) was used for all the studies. Octave can be installed in both Windows OS as well as open source Ubuntu OS enabling a completely open source possibility. The Octave codes with comments for phase retrieval is provided in Supplementary Material 4. The Octave codes for frame grabbing [45] using a web camera is provided in Supplementary Material 5.

**Figure 7:**
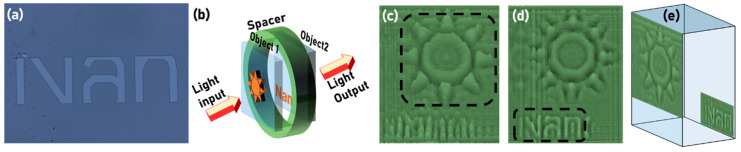
(**a**) Optical microscope image of the fabricated object. (**b**) Thick object construction using two thin objects. Phase reconstruction results for (**c**) star object and (**d**) ‘Nan’ object. (**e**) 3D QPI reconstruction.

From the colour bar shown in Figure 6, it is seen that the phase difference between the two levels is approximately π, which corresponds to a thickness of 530 nm, which is lesser than the measured value of 800 nm. The difference between the retrieved phase and experimental value is partly due to the broader spectral width of 33 nm, and partly due to experimental and computational errors. The results shown in Figure 6 are phase maps and images for a single axial plane and therefore does not constitute three dimensional QPI. To demonstrate QPI in 3D, thick objects were constructed by attaching two different thin phase objects with a 3 cm spacer. A phase object with a negative resist profile ‘Nan’ was fabricated using EBL, as shown in Figure 7a, and the thick object construction is shown in Figure 7b. The phase image reconstructions at the two planes are shown in Figure 7c,d respectively. In Figure 7c, the star object is in focus, while in Figure 7d, the ‘Nan’ object is in focus. The phase retrieval algorithm was executed with a different distance *d* + 3 cm, to obtain the reconstruction for the second plane. The combined phase reconstruction is shown in Figure 7e.

## 6. Discussion

The phase difference step of imaged objects (in air) was given as 2π*t*(*n* − 1)/λ, which is approximately 1.5π for PMMA of refractive index 1.5 and thickness 800 nm at 530 nm wavelength. This is a small phase change typical for bio-imaging microscopy carried out in solution (refractive index 1.33 of water) and for micro optical elements. The lateral resolution of imaging is limited by the pixel size of the camera rather than the NA of the lens, as in the case of regular microscopy. The field of view is limited by the physical size of the image sensor. There is some degree of freedom in the lensless case, with a diverging wave illumination of the sample, where the distance *d*_2_ can be adjusted to trade-off resolution of imaging with the field of view. The phase retrieval algorithm required as low as only 2 iterations to converge and the execution time was <2 s in a computer (Intel Core i5-8250U CPU @ 1.60 GHz and 1.80 GHz). It must be noted that the limitation will not apply for real time observation of a biological event or fabrication of a micro optical element, unless the event needs a real time intervention.

## 7. Summary, Conclusions, Outlook

A tutorial for a compact, single camera shot, three-dimensional QPI technique using a partially coherent light source has been demonstrated with pseudocodes and Octave codes. The tutorial uses a set of low-cost and easily available components: low cost LEDs, basic camera or web camera, open source OS and open source Octave software, with spatial and temporal resolutions on par with regular microscopes, but with a cost at least an order lower than conventional microscopes. The supplementary files for automating web camera and quantitative phase imaging using Octave has been provided. This integrated approach creates a viable tool for 3D QPI for the real-time observation of biological events. The technique has been demonstrated for only two planes. Consequently, the proposed method can be used to monitor multiple planes of an object simultaneously, in real time, a capability only present in 3D phase scopes. The phase retrieval algorithm is highly sensitive to various optical and computational parameters, such as distances, external light, sampling, zero padding, object size, initial guess phase and amplitude patterns, etc. Once an optical system and the algorithm is calibrated to achieve optimal reconstruction, the method can be effective. One of the main drawbacks of using a source with a larger spectral width is the uncertainties present in the determination of phase values. While the contrast within 0–2π cycle is relatively lower than the original phase pattern, it is possible to measure phase profiles beyond the 0–2π range and unwrap it to the original phase without modulo-2π folding.

This method is promising for bio-microscopy of cells and particularly for monitoring the interaction of bio-membrane with the nanotextured surface to reveal mechano-bactericidal action [46]. There is a strong application potential of the QPI method in laser tweezers [47], where optically transparent 3D objects are manipulated in complex environments with different reflectivities and shapes of micro-surroundings. Modifications inside transparent materials, phase changes and refractive index alterations during femtosecond laser writing of micro-optical elements [44] can be augmented with in situ monitoring using the proposed method. These are directions that will be explored in the application of this QPI. The technique can also be integrated into polariscopy for orientation measurements [48]. We believe that the tutorial will be useful for beginners and young scientists who would like to begin research in the area of QPI and follow the latest developments in the area [49,50]. Furthermore, the low-cost and easy to implement conditions will encourage academic research activities on QPI in developing countries.

## Figures and Tables

**Table 1:** Pseudocode for Phase retrieval algorithm.

Task. No	Task	Steps
1	Defining Computational space	Step-I Define the length and breadth of the computational space in pixels (2×*N*_1_, 2×*N*_2_). Step-II Define origin (0, 0), *x* and *y* coordinates: *x* = (−*N*_1_ to *N*_1_ − 1), *y* = (*N*_2_ to *N*_2_ − 1). Step-III Define pixel size Δ and wavelength *λ* (pixel = camera pixel size, lambda). Step-IV Create meshgrid: (*X*, *Y*) = meshgrid (*x*×pixel, *y*×pixel).
2	Defining initial matrices and forward and backward propagators	Initial matrices: *Sensor plane*-Amplitude *A*_1_ = 0 (for all *X*, *Y*) and *A*_1_ (*N*_1_/2:3*N*_1_/2 − 1, *N*_2_/2:3*N*_2_/2 − 1) = *I*^1/2^, where I is the normalized recorded intensity pattern and phase *P*_1_ = 0 (for all *X*, *Y*). *Sample plane*-Amplitude *A*_1_ = 1 (for all *X*, *Y*). Propagators: *Forward propagator*: S[R(d)]=exp[j2πR(d)/λ]. *Backward propagator*: S[−R(d)]=exp[−j2πR(d)/λ]. where $R\left(d\right)={\left(X^{2}+Y^{2}+d^{2}\right)}^{1/2}$.
3	Phase retrieval	Construct the initial complex amplitude *C*_1_ at the sensor plane as *C*_1_ = *A*_1_ exp(*jP*_1_). Start *for* loop Step-I Convolve the initial complex amplitude with the backward propagator: $C_{2}={ℑ}^{-1}\left[ℑ\left\{S\left[-R\left(d\right)\right]\right\}\times ℑ\left\{C_{1}\right\}\right]$. Step-II Replace the amplitude of *C*_2_ with *A*_2_ and carry-on the phase *P*_2_ at the sample plane i.e., *C*_2_ = *A*_2_ exp(*jP*_2_). Step-III Convolve the modified complex amplitude *C*_2_ with the forward propagator: $C_{1}={ℑ}^{-1}\left[ℑ\left\{S\left[R\left(d\right)\right]\right\}\times ℑ\left\{C_{2}\right\}\right]$. Step-IV Replace the amplitude of *C*_1_ by *A*_1_ and carry on the phase for the next iteration. Iterate Steps I–IV until the phase pattern is generated with a minimum error indicated by the convergence of the correlation co-efficient *C* (*x* = 0, *y* = 0) to a stable value. Display *P*_2_. End *for* loop

## Supplementary Materials

The following are available online at https://www.mdpi.com/2313-433X/6/9/99/s1, Supplementary Materials (1–5).
